# Numerical Simulation of High Strain Rate and Temperature Properties of Laser Powder Bed Fusion Ti6Al4V(ELI) Determined Using a Split Hopkinson Pressure Bar

**DOI:** 10.3390/ma15051872

**Published:** 2022-03-02

**Authors:** Amos Muiruri, Maina Maringa, Willie du Preez

**Affiliations:** 1 Department of Mechanical and Mechatronics Engineering, Central University of Technology, Free State, Bloemfontein 9301, South Africa; mmaringa@cut.ac.za; 2 Centre for Rapid Prototyping and Manufacturing, Faculty of Engineering, Built Environment and Information Technology, Central University of Technology, Free State, Bloemfontein 9301, South Africa; wdupreez@cut.ac.za

**Keywords:** SHPB, Ti6Al4V(ELI), VUMAT, VUHARD, additive manufacturing, numerical modelling

## Abstract

Numerical models can be useful for analysis of the ability of structural engineering materials to withstand harsh environmental conditions such as dynamic loading. In the present study, a microstructure-variable-based numerical model for predicting the high strain rate and temperature properties of different microstructures of Ti6Al4V (ELI-Extra Low Interstitial) produced by laser-based powder bed fusion is proposed. The model was implemented in two different subroutines, VUMAT and VUHARD, available in ABAQUS/Explicit for simulating dynamic conditions. The two subroutines were then used to simulate the split Hopkinson pressure bar (SHPB) experiments to study the flow properties of various forms of the direct metal laser sintered Ti6Al4V(ELI) alloy at various conditions of strain rate and temperature. Comparison of the results obtained through simulation and those obtained from experimental testing showed high degrees of correlation and accuracy with correlation coefficients and absolute percentage errors >0.97 and <4%, respectively. The numerical model was also shown to give good predictions of the strain hardening and dynamic recovery phenomena that prevail for deformations at high strain rates and temperatures.

## 1. Introduction

Among several metal additive manufacturing (AM) technologies available today, the powder bed fusion (PBF) process is the most preferred by industries because of the relatively good surface finish and accuracy that it can achieve [1]. Research over the last few decades and industrial advancement have enabled a good uptake of commercial applications of the PBF process in manufacturing of high-value products that are burdensome to produce by conventional manufacturing processes [2]. PBF processes are classified according to the source of heat such as laser and electron beam and type of powder feed. Direct metal laser sintering (DMLS) is among the laser-based powder-bed methods that are currently being explored to produce high-value 3D metallic functional components ready to be used in biomedical and aircraft industries [3,4]. Various benefits of this manufacturing technology are being realized by these industries. Improvement in the freedom of design, weight reduction due to production of lattices and precise internal hollow structures, monolithic designs that replace assembly of multiple components and design customisation are among the gains of this technology [5].

Currently, there are no specific limitations that state which component can or cannot be produced by additive manufacturing (AM) for use in demanding structural applications such as in aircraft engines. Material scientists and engineers in academic and industrial research centres around the globe are working to determine where commercial opportunities for AM exist. Lightweight structural metals and alloys are the most popular materials for AM processes due to their many uses in aircraft. Thus, several different metals and their alloys are available in powdered form for PBF process requirements. These include titanium alloys, aluminium alloys, nickel-based alloys and stainless steel. The Ti6Al4V is the most widely used alloy of titanium and is commonly utilized as material for aircraft gas turbines and airframes because of its high specific strength, excellent fatigue strength, high fracture toughness and crack propagation resistance [6]. Its high corrosion resistance and biocompatibility also make it attractive for use in manufacturing biomedical implants [7] and for use in chemical and marine industries [8]. Due to this wide usage of Ti6Al4V, it is one of the most common metallic materials employed in AM.

The mechanical properties of AM-produced and postprocessed (surface polished, heat treated and hot isostatic pressed) Ti6Al4V parts have been studied widely and the data obtained published in open literature [9,10,11,12,13,14,15,16,17,18,19,20,21,22,23]. Mechanical properties of as-built AM Ti6Al4V subjected to surface mechanical posttreatment were reported in [9]. The cyclic plasticity and low- and high-cycle fatigue properties of DMLS Ti6Al4V have been reported in various research works [10,11,12,13]. Various studies have reported the tensile properties of as-built, as-built and hot isostatically pressed and annealed AM Ti6Al4V [12,14,15,16]. The impact toughness of as-built and stress relieved DMLS Ti6Al4V (Extra low interstitial-ELI) was reported in [17,18]. Other researchers have studied the quasi-static and high-strain-rate behaviour of AM Ti6Al4V parts [19,20,21,22,23]. The known mechanical properties of a material are an integral part of any design and manufacturing process and serve to validate the suitability and performance of the final products. However, mechanical properties obtained from test specimens do not always represent the performance of real components and structures since they vary as a function of factors such as design shape, dimensions and the point of application of load [24].

High-strain-rate properties are essential in the development of material for use under strong dynamic conditions. Dynamic conditions are dominant in aircraft during flight phases such as landing and incidences such as bird strike, foreign object damage (FOD) and fan blades out (FBO) events [25,26,27]. The split Hopkinson pressure bar (SHPB) test, developed by Bertram Hopkinson [28] and later modified and refined by Kolsky [29] is widely employed to investigate the dynamic behaviour of materials. The method is used to obtain the stress-strain curves of materials at strain rates above 10^2^ s^−1^ [30]. However, the SHPB test is sophisticated and very expensive, and the accuracy of the obtained results depend on several factors such as data processing, test conditions and material response [31]. Thus, the demand for the use of constitutive numerical modelling and simulation has increased with the advancement of modern technology.

Numerical simulations of the behaviour of materials require constitutive equations that account for properties such as inelastic deformation (flow stress) and damage evolution as a function of variables such as strain, strain rate and temperature. Because of simplicity and the wide availability of required coefficients and constant parameters, numerical simulations of dynamic properties of materials such as alloys of titanium, aluminium, copper and steel, are normally based on the Johnson–Cook (J–C) material model [32,33,34,35]. This model is in-built in the commercial finite element analysis (FEA) software ABAQUS/Explicit for simulating high-strain-rate deformation of materials including the simulation of adiabatic transients [36]. The drawback of the J–C model is that it shows large inconsistencies between various sets of calibrated parameters [37]. This has been suggested to be a result of differences in the microstructure of the alloy. For instance, the difference in the relative amounts of α and β-phases present in the Ti6Al4V alloy has been found to influence the calculated coefficient and constant parameters of the J–C model [38]. Yu et al. [39] also found that the Ti6Al4V alloy exhibits completely different sets of J–C model parameters as a function of the type of microstructure (equiaxed, lamellar or bimodal), the volume fraction of α-phase and the thickness of α-grains. It is essential therefore, to develop a more robust and reliable numerical model that can adequately tailor the microstructure of materials to its mechanical properties for a wide range of external state variables. Such a numerical model would be easier to use and more reliable for use in making design modifications without having to expend time and expenses that come with experimental test iterations and the manufacture of multiple prototypes. Such models can also be useful in carrying out common aircraft dynamic simulations involving large plastic deformation such as dynamic response analysis of bird strikes on aircraft [40], fan blade off (FBO) events and impact resistance of turbo-engine containment structures [41].

This paper documents the validation of a numerical model based on a microstructural-variable-based constituted model for simulating the SHPB experimental results of high-strain-rate and temperature properties of various forms of DMLS Ti6Al4V(ELI). The microstructural-variable-based constitutive model was developed and calibrated in the authors’ previous work [42] based on the average grain sizes and the density of dislocations in different microstructures of the DMLS Ti6Al4V(ELI) alloy. The numerical model validated here was first implemented as two different subroutines, VUHARD and VUMAT available in ABAQUS/Explicit for dynamic simulations, and its accuracy tested and verified using single and multiple element meshes and various loading conditions. The results of this verification can be found in the authors’ previous work in Muiruri et al. [43]. In this paper the SHPB numerical model is built to verify the accuracy of the two-subroutines implemented in ABAQUS/Explicit in predicting the high-strain-rate properties of various forms of DMLS Ti6Al4V(ELI). The simulation was carried out under the same conditions as the SHPB experimental tests performed on the three different forms of this alloy, with the results published in [44]. Comparison between the SHPB numerical and experimental results for different forms of DMLS Ti6Al4V(ELI) showed a high degree of correlation with a correlation coefficient above 0.97 and an absolute percentage error below 4%.

## 2. The Principle of the SHPB Experiment

**Figure 1:**
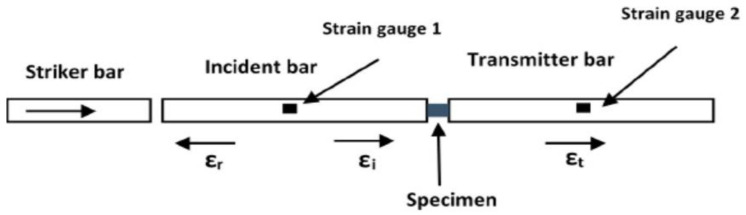
Schematic diagram illustrating the compression SHPB experimental setup.

The SHPB apparatus is commonly applied in uniaxial loading configurations in compression, tension and torsion high-strain-rate tests [30,31]. It consists of the incident/input bar, the transmitter/output bar and the striker bar. The conventional compression SHPB apparatus which is of interest in this study is illustrated in Figure 1.

In this figure symbols $\varepsilon_{i}$, $\varepsilon_{r}$ and $\varepsilon_{t}$ denote the incident, transmitted and reflected waves. The incident, transmitter and striker bars are usually made of the same material and are mostly of the same cross-sectional area. The test specimen is normally sandwiched between the incident and transmitter bars. During testing, the striker bar shown in Figure 1 is fired from a gas gun/cannon and impacts the free end of the incident bar at a high velocity. The impact of the striker bar onto the input bar produces a compressive wave (incident wave) travelling toward the specimen. Upon reaching the specimen, the incident wave becomes partially reflected due to an impedance mismatch of the bar-specimen interface and partially transmitted through the transmitter bar. Continuous reverberation of reflected waves occurs across the length of the test specimen until the stress build-up is sufficient to cause plastic deformation [30]. The strain gauges shown in Figure 1 are used to measure the strain in each of the two bars. Using the theory of one-dimensional elastic wave propagation in a cylindrical specimen, the measured strain data is used to determine the stress, strain and strain rate acting on the test specimen using the following equations [30,31]:

(1) $$\sigma_{s}=EA_{0}\varepsilon_{t}/A_{s}$$

(2) $$\varepsilon_{s}=2C_{0}/l_{s}\int_{0}^{t}\varepsilon_{r}dt$$

(3) $$\dot{\varepsilon_{s}}=2\varepsilon_{r}C_{0}/l_{s}$$

where the symbols $A_{0}$, $A_{s}$, $l_{s}$, $C_{0}$, E and t denote the cross-sectional area of the bars, initial cross-sectional area of the specimen, length of the specimen, wave velocity of the bars, elastic modulus of the bars and time, respectively.

## 3. A Microstructural-Variable-Based Model for High-Strain-Rate Flow Properties of AM Ti6Al4V(ELI)

The titanium alloy, Ti6Al4V, exhibits different sets of mechanical properties arising from the modification of its microstructure by different heat treatment cycles. It is generally a complex dual-phase alloy, and its microstructural features are dependent on length scales in a way that strongly influences its mechanical properties. It is therefore imperative to control the microstructure of the heat treated DMLS Ti6Al4V(ELI) to obtain the desired mechanical properties for a given desired functional structure. Numerical modelling is a powerful method that can be used to predict the mechanical properties of various microstructures of this DMLS alloy in lieu of many expensive experimental tests required to obtain the corresponding data. A microstructural-variable-based constitutive equation that can model the mechanical properties of DMLS Ti6Al4V(ELI) for a wide range of external state variables was developed in Muiruri et al. [42]. In this model the total flow stress (σ) is of the form:

(4) $$\sigma =\sigma_{mts}\left(\dot{\varepsilon },T\right)+\sigma_{t}\left(t\right)+\sigma_{p}\left(\varepsilon_{p},T,\rho \right)+\sigma_{drag}\left(\dot{\varepsilon }\right)$$

The first term of this equation ($\sigma_{mts}$) denotes the mechanical threshold stress (MTS) model, which is usually a function of strain rate ($\dot{\varepsilon }$) and temperature (*T*), and is of the form [42,45,46]:

(5) σmts=μTμ0σo1−kbTg0iμTb3ln ε˙oε˙1q1p

where the symbols $\sigma^{o}$, $k_{b}$, b, $\dot{\varepsilon }$, ${\dot{\varepsilon }}_{o}$ and $g_{0i}$ are the values of thermal stress at −273 °C, Boltzmann’s constant, Burgers vector, testing strain rate, reference strain rate and material constant, respectively. The constants p and q are fitting parameters of the MTS model. The symbol μ denotes the shear modulus, which is a function of temperature, T, while $\mu_{o}$ is the value of the shear modulus of a material at −273 °C. The second term ($\sigma_{t}$) in Equation (4) is the Hall–Petch relationship that describes the inverse relationship between the grain sizes (α-laths thicknesses ($a_{t}$)) in case of Ti6Al4V with lamellar microstructure) and yield stress of a material with a given microstructure as [47]:

(6) $$\sigma_{t}=\frac{K_{H-P}}{\sqrt{a_{t}}}$$

where symbol $K_{H-P}$ denotes the Hall–Petch constant. The third part of Equation (4) ($\sigma_{p}$) denotes the integrated Taylor hardening law that describes the evolution of dislocation density (ρ) with plastic strain ($\varepsilon_{p}$) and is of the form [42,48]:

(7) $$\sigma_{p}=\alpha \mu \left(T\right)bM{\left(\frac{h}{k_{2}}\;\left(1-\exp\left(-k_{2}\varepsilon_{p\;}\right)\right)+\rho_{o}\exp\left(-k_{2}\varepsilon_{p}\right)\right)}^{\frac{1}{2}}$$

where symbols α, b, M and $\rho_{o}$ are a material constant, Burgers vector, Taylor factor and initial dislocation density in a material. The parameters h and $k_{2}$ are the coefficients of dislocation accumulation and annihilation, respectively.

The final part of Equation (4) represents the viscous drag stress ($\sigma_{drag}$) that describes the upturn of the flow stress of a material at strain rates (${\dot{\varepsilon }}_{p}$) above 10^3^ s^−1^ and is of the form [49,50]:

(8) $$\sigma_{drag}=\zeta .\left(1-exp\left(-\chi \;.{\dot{\varepsilon }}_{p}\right)\right)$$

where symbols ζ and χ are model calibration parameters.

## 4. Materials and Methods

### 4.1. Production and Heat Treatment of DMLS Ti6Al4V(ELI) Alloy

The high-strain-rate test specimens were produced from pre-alloyed Ti6Al4V(ELI) (grade 23) fine powder by the DMLS process using an EOSINT M280 machine. The powder particles were spherical in shape with an average diameter less than 40 µm (D_10_, D_50_, D_90_), while the chemical composition in wt.% of the powder as provided by TLS GmbH is shown in Table 1 [51], which complies with the ASTM F3001-14 standard [52].

**Figure 2:**
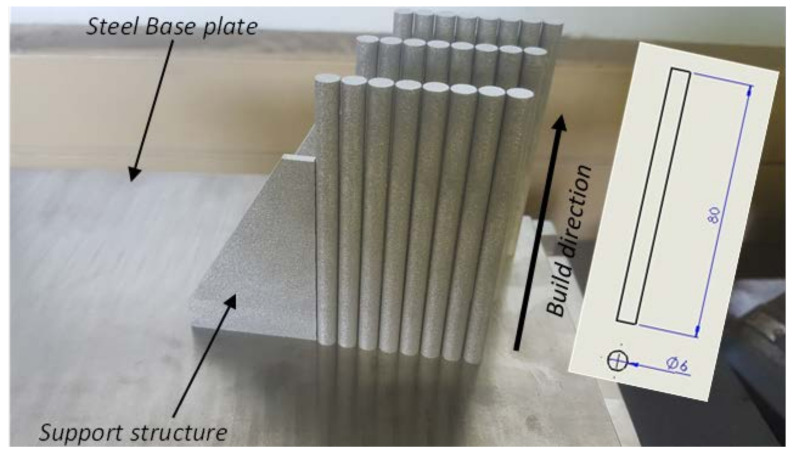
The manufactured SHPB test specimens on DMLS E0SINT M280 machine build platform. The geometrical specifications in mm are shown in the insert of the figure.

The process parameters of the EOSINT M280 machine used in this work *viz*. laser power setting, laser diameter, hatch spacing, layer thickness and scanning speed were 175 W, 80 µm, 100 µm, 30 µm and 1400 mm/s, respectively. A total of 24 cylindrical specimens shown in Figure 2, each with diameter and length of 6 mm and 80 mm, respectively, were produced.

The manufactured specimens shown in Figure 2 were first stress-relieved in a vacuum furnace before being cut off from the steel base plate and later subdivided into three groups designated hereafter as samples C, D and E for further high temperature annealing. The stress relieving heat treatment was executed at a temperature of 650 °C, with a residence time of 3 h, and subsequently the samples were furnace cooled to room temperature. Cutting was executed using an electric discharge machine (EDM-wire cutter). The purpose of heat treatment in this research work was to obtain different microstructural features of the alloy with variable mechanical properties. Samples C were annealed at 800 °C for a residence period of 2.5 h then furnace-cooled to room temperature. Samples designated D were duplex-annealed at 940 °C for 2 h then furnace-cooled, followed by heat treatment at 750 °C for 2 h, then finally furnace-cooled. Samples designated E were heat treated above the β-transformation temperature at 1020 °C for 2.5 h before being furnace-cooled to room temperature. The analysis of the microstructures obtained after each heat treatment cycle described here can be found in Muiruri et al. [53,54]. The average grain size and initial dislocation density of a microstructure are part of the input parameters of the model presented in Section 3. The average size of α-laths for the microstructures of samples C, D and E was determined by the line intercept method and the results reported in Muiruri et al. [44,53], while the average initial dislocation density in these samples was determined by the method of X-ray diffraction and the results reported in [53]. A summary of these critical model input parameters presented in [44,53,54] are presented in Table 2.

### 4.2. SHPB Experimental Tests

The SHPB tests were designed to generate the stress-strain curves of samples C, D and E at different high strain rates and temperatures. Such flow stress curves were used for validation and verification of the numerical model. The pressure bars used for these tests were made of tool steel and were 2000 mm long with a diameter of 20 mm. The striker bar was made of the same material and was 500 mm long and the same diameter as the pressure bars. The test specimens were cut off from samples C, D and E. The SHPB specimens had a diameter and height of 6 mm. The ends of these specimens were faced off using a lathe machine to ensure that they were flat and parallel to achieve the best possible contact between specimen and pressure bars. To minimize friction and therefore maintain a uniaxial state of compression, lithium based NLGI3 grease (Castrol WB) was used to lubricate the interface between the pressure bars and the test specimen.

To carry out the SHPB tests at high temperature, the specimens were heated to a particular temperature in a furnace and then brought into contact with the bars a short moment before impact. The drawback of this approach was the duration of time (≈8 sec), during which the test specimens were in contact with the pressure bars before arrival of the incident wave as this led to a reduction of the test temperature of the specimen. Thus, to compensate for any drop of temperature by the specimen during this time before impact, the test specimens were soaked at a temperature 30 °C higher than the desired test temperature. 

The SHPB equipment used for the study was only limited to a strain rate of about 3000 s^−1^ for an impact velocity of about 25 m/s. Thus, the samples were tested at three different impact velocities of 8 m/s, 15 m/s and 25 m/s generated by firing the striker bar at pressures of 4 bars, 7 bars and 13 bars, respectively. The test at each of these impact velocities was conducted at three different temperatures of 25 °C, 200 °C and 500 °C. The maximum test temperature selected for this study was informed by the maximum allowable temperature for Ti6Al4V (less than 500 °C) to avoid causing microstructural transformation during heating. The obtained flow stress curves for samples C, D and E at different high strain rates and temperatures can be found in [44]. These curves were used to refine and calibrate the model presented in Section 3 as reported in [42]. A summary of the numerical model input parameters obtained in the present work together with other parameters found from other sources is shown in Table 3.

## 5. Implementation of the Constitutive Model as Material Subroutine in Abaqus

The analytical constitutive model presented in Section 3, was implemented separately in two ABAQUS/Explicit VUHARD and VUMAT subroutines available for defining the yield surface and isotropic plasticity of materials. The implementation process, testing and verification of these two subroutines can be found in the authors’ previous work in Muiruri et al. [43]. The VUMAT subroutine is used to describe the mechanical elastic-plastic constitutive behaviour of materials while the VUHARD subroutine only takes care of the inelastic part of a material’s behaviour. These two subroutines, therefore, have different implementation procedures and the accuracy of each subroutine in the simulation of SHPB experiments was tested in this research.

## 6. The SHPB Finite Element Model

The 3D finite element model (FEM) for the SHPB setup used for this study consisted of the incident, transmitter and striker bars modelled with tool steel. The geometry and dimensions of these modelled bars were as those of the experimental test described in Section 4.2. The Poisson’s ratio, elastic modulus and the density assigned to these bars were 0.3, 230 GPa and 8050 Kg/m^3^, respectively [61]. Cylindrical test specimens of DMLS Ti6Al4V(ELI), with length and diameter of 6 mm were used throughout the simulation. The flow stress curves of these specimens were modelled using the constitutive numerical material models implemented in VUMAT and VUHARD presented in the authors’ previous work found in [43].

A “hard” contact that allows separation after contact was used as the normal interaction property between the specimen and the bars on either side of it. The “hard” contact relationship in ABAQUS is normally used to obviate penetration of one surface into another at locations of contact [36,62]. This provision in the simulation of the SHPB test between the specimens and adjacent bars was used in the present work to ensure that neither the specimens nor bars penetrated one another upon impact. 

For the SHPB experiment to be valid, there must be minimum friction and inertial effects between the specimen and the pressure bars. This is normally achieved by applying a lubricant at the interfaces of the specimen and bars [30,31]. Friction between the specimen and the bars is unwanted during the SHPB compression to minimise radial constraint that could introduce a nonuniform triaxial stress state in the specimen. Thus, the tangential behaviour at the interface between the specimen and bars on either side of it was modelled as frictionless in all simulations. 

**Figure 3:**
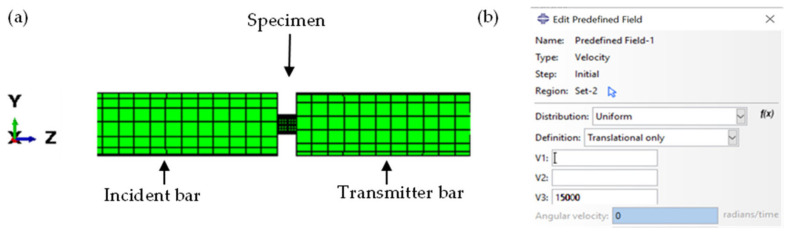
(**a**) A longitudinal section of a numerical model for the SHPB test and (**b**) the velocity (expressed in mm/s) in the predefined field imposed along the z-axis.

The classical compressive SHPB set up is made up of mechanical supports with bearings that provide accurate uniaxial alignment and free axial motion of the bars while preventing transverse motion of the loading axis. Since the SHPB equipment works on the principle of one-dimensional wave propagation [30,31], constraints were applied to the bars to allow the propagation of stress waves only in the z-direction. This was achieved by applying the displacement type of initial boundary conditions that prevent transverse motion of the pressure bars (U1 and U2), thus allowing the displacement of bars only along the z-direction (U3). Figure 3 shows a longitudinal section of the components of the numerical model of the SHPB test.

**Figure 4:**
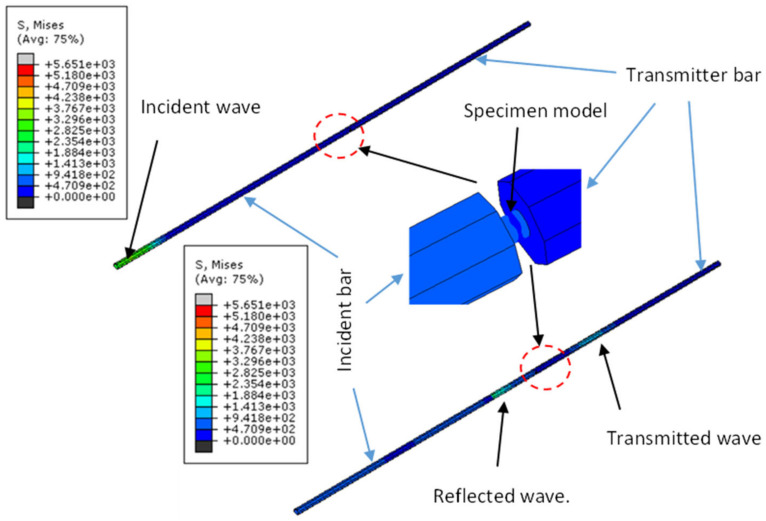
Numerical simulation of a compressive wave in a SHPB test.

A uniform velocity was applied to the nodes of the striker bar to yield impact loading in ABAQUS/Explicit. This velocity was set in the predefined field in ABAQUS shown in Figure 3b. During the simulation, the launched striker bar strikes the input bar with a pre-determined velocity to induce a desired strain rate. The incident wave in the incident bar at the beginning of the simulation and the transmitted and reflected waves at the transmitter and incident bars, respectively, just after the induced stress wave passes the specimen, are shown in Figure 4.

## 7. Results and Discussion 

### 7.1. Mesh Convergence Analysis

**Figure 5:**
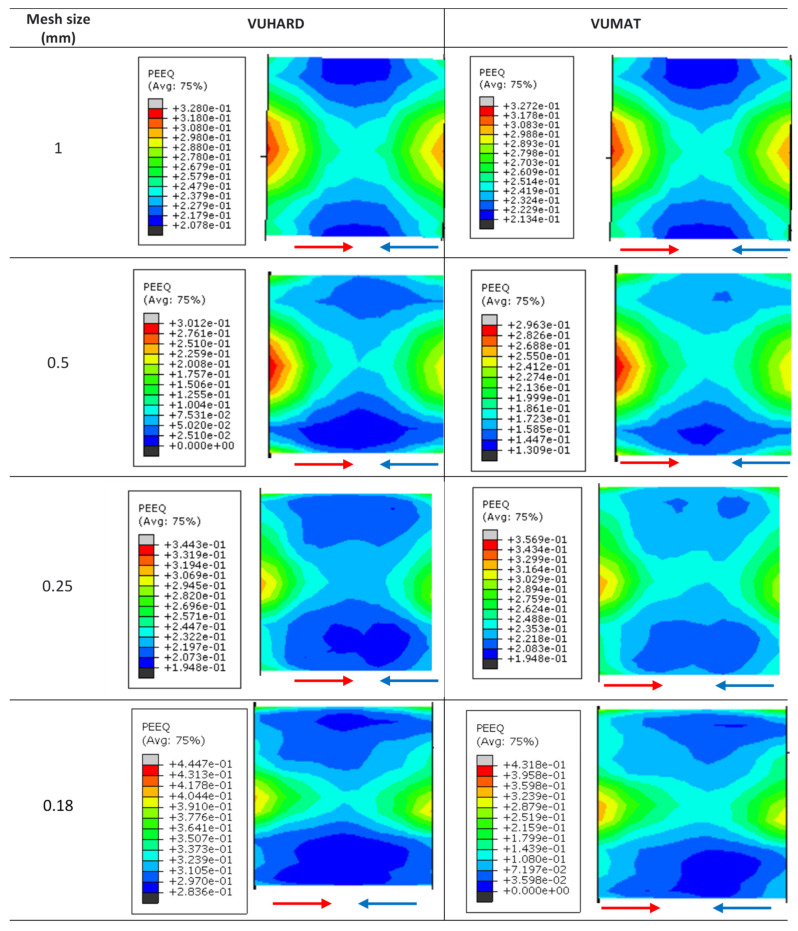
The equivalent plastic strain contours for a simulated SHPB test at a striker velocity of 8 m/s for 1 mm, 0.5 mm, 0.25 mm and 0.18 mm mesh sizes. (The red and blue arrows indicate the direction of incident and reflected waves, respectively.)

**Figure 6:**
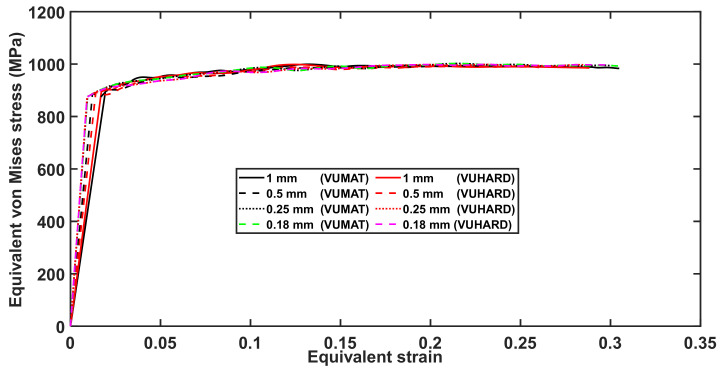
Curves of the numerical equivalent von Mises stress against plastic strain at a temperature of 25 °C and imposed velocity of 8 m/s for different mesh sizes in numerical simulation of the SHPB test using the VUMAT and VUHARD subroutines developed in the present work.

A mesh convergence study was first carried out with the proposed VUMAT and VUHARD models to find the optimum mesh density to give the most accurate results in the simulation of the dynamic properties of DMLS Ti6Al4V(ELI) using the SHPB test. Continuum elements C3D8R were used which have meshes with global sizes of 1 mm, 0.5 mm, 0.25 mm and 0.18 mm. The mesh convergence study was carried out by imposing an impact velocity of 8 m/s through the striker bar at a simulation temperature of 25 °C. The corresponding arising equivalent plastic strain contours are shown in Figure 5, while the stress-strain curves resulting from the simulation using the four mesh sizes are shown in Figure 6. The summary of the computation time in the SHPB test simulations using different mesh sizes is presented in Table 4.

The equivalent plastic strain profiles for the SHPB test specimen are seen in Figure 5 to be almost the same for all four mesh sizes. The maximum equivalent plastic strain on the test specimen is seen to be located at the surfaces in contact with the incident and transmission bars, and a distinct strain zone that forms an “X-shape” pattern through the diameter of the deformed model sample is visible in all cases.

The VUMAT subroutine is seen in Table 4 to require more computation time compared to the VUHARD subroutine. As expected, the computation time increases as the mesh size decreases. It is seen from Figure 6 that the stress-strain curves for various mesh sizes nearly overlap for the better part of the profiles, and the curves for the 0.25 mm and 0.18 mm mesh sizes are indistinguishable. It should be noted here that extensive mesh convergence analysis for the simulation of multiple element mesh models using the implemented VUMAT and VUHARD subroutine were carried out in Muiruri et al. [43]. The researchers in the referred study found that the values of equivalent plastic strain and von Mises stress converged for mesh sizes less than 0.5 mm.

The effect of mesh sizes in Figure 6 is seen to be significant at the initiation of equivalent plastic strain, which is consistent with the observations also made in Muiruri et al. [43]. It is seen in this figure that the value of initial plastic strain increases with the increase in mesh size. These initial values of strain are very small, thus the difference in computed initial von Mises stress from these strain values is 1.7% between 1 mm and 0.18 mm mesh sizes and 0.87% between 0.5 mm and 0.18 mm mesh sizes. Considering the computation time shown in Table 4 and the observation made with reference to Figure 6 and in [43], it can be concluded that 0.25 mm mesh size is sufficient to obtain a good prediction. This is the case, since a smaller mesh size would show a negligible improvement of the stress-strain curve and require more computation resources. Therefore, all ensuing simulations to generate stress-strain curves for various forms of DMLS Ti6Al4V(ELI) were conducted with a 0.25 mm mesh size in the present study.

### 7.2. The SHPB Simulation Test Results and Discussion

**Figure 7:**
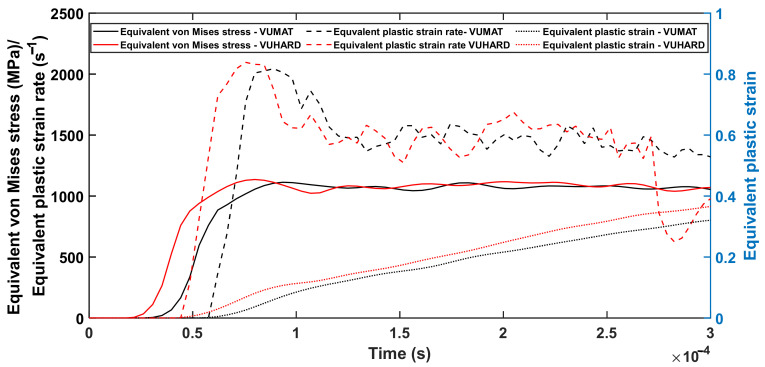
The equivalent von Mises stress, plastic strain and plastic strain rate against time generated during the simulation of the SHPB test using the VUMAT and VUHARD subroutines at an imposed striker velocity of 15 m/s and at a temperature of 25 °C.

Simulations were carried out with striker velocities of 8 m/s, 15 m/s and 25 m/s at temperatures of 25 °C, 200 °C and 500 °C following the experimental setup described in Section 4.2. To ensure sufficient plastic deformation and at the same time avoid excessive distortion of the model, different total step times of 1000 µs, 300 µs and 200 µs for the velocities of 8 m/s, 15 m/s and 25 m/s, respectively, were used. Typical curves of equivalent plastic strain, plastic strain rate and von Mises stress against simulation time at a velocity of 15 m/s are shown in Figure 7.

**Figure 8:**
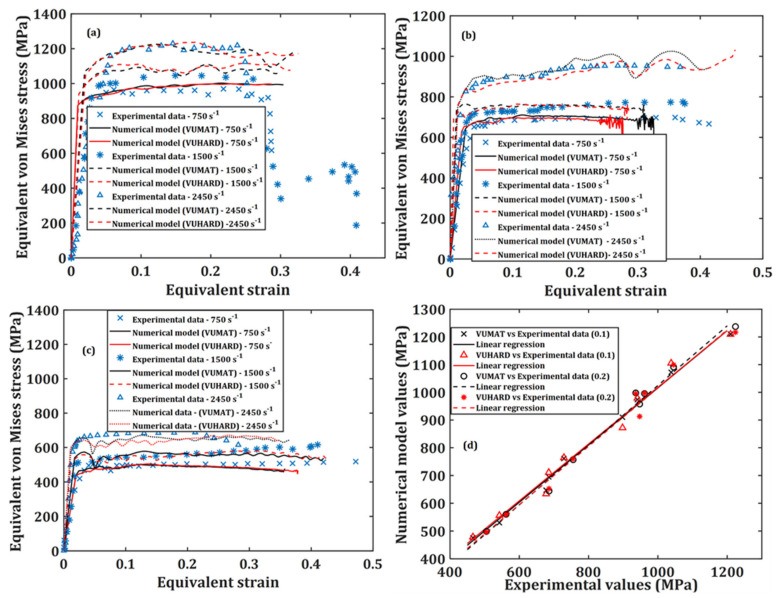
Graphs of the numerical model prediction and the experimental results for samples C, at three high strain rates and at temperatures of (**a**) 25 °C; (**b**) 200 °C and (**c**) 500 °C; and correlation between the numerically predicted values and those obtained from experimentation at strains of 0.1 and 0.2 in (**d**).

**Figure 9:**
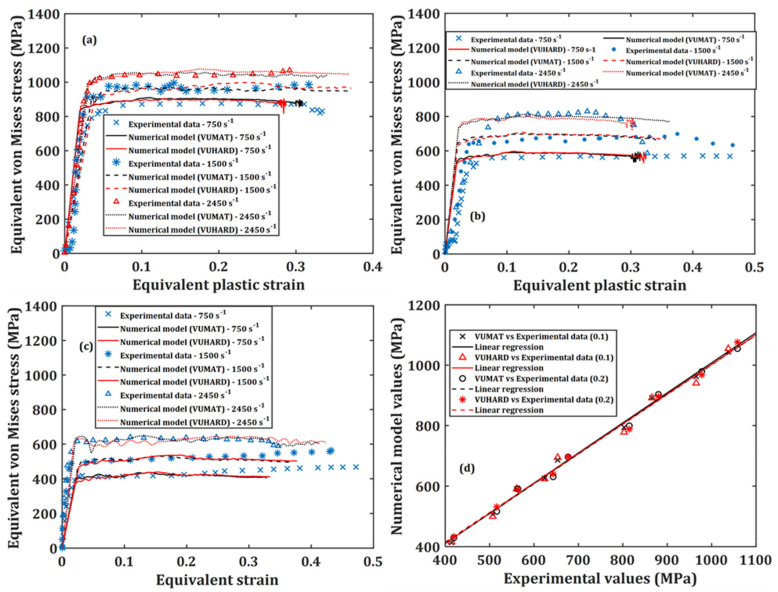
Graphs of the numerical model prediction and the experimental results for samples D, at three high strain rates and at temperatures of (**a**) 25 °C; (**b**) 200 °C and (**c**) 500 °C; and correlation between the numerically predicted values and those obtained from experimentation at strains of 0.1 and 0.2 in (**d**).

**Figure 10:**
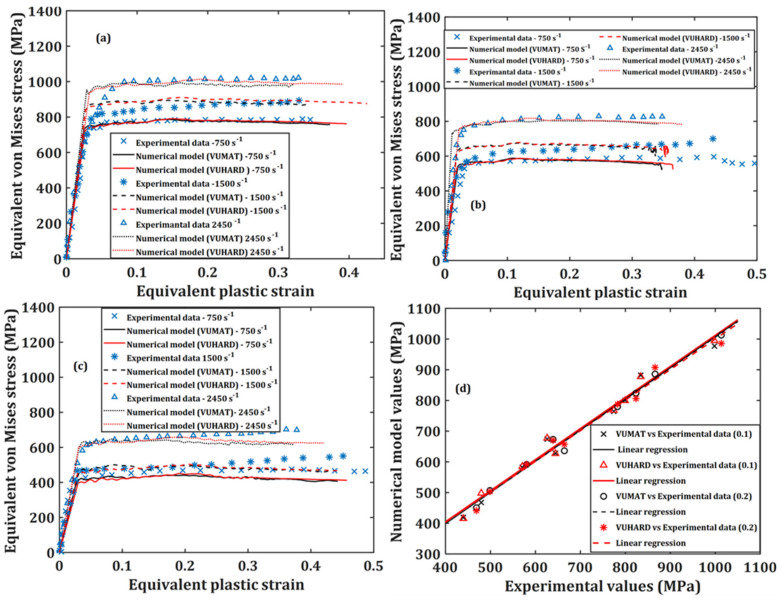
Graphs of the numerical model prediction and the experimental results for samples E, at three high strain rates and at temperatures of (**a**) 25 °C; (**b**) 200 °C and (**c**) 500 °C; and correlation between the numerically predicted values and those obtained from experimentation at strains of 0.1 and 0.2 in (**d**).

From Figure 7, the model is seen to have experienced near linearly increasing strains over most of the period of strain buildup. During this period, the simulation results eventually averaged at an equivalent plastic strain rate of about 1500 s^−1^, a value similar to that recorded during the experimental results reported in [44]. This demonstrates great confidence in the SHPB numerical model setup and the implemented subroutines developed in this work. Figure 8, Figure 9 and Figure 10 show a comparison between the experimental results and results from the numerical model at average plastic strain rates of approximately 750 s^−1^, 1500 s^−1^ and 2450 s^−1^ and temperatures of 25 °C, 200 °C and 500 °C. The numerically predicted values at the equivalent strain of 0.1 and 0.2 for the same test conditions were also plotted against one another and are presented in (d) of Figure 8, Figure 9 and Figure 10. 

The ability of the implemented numerical model in the VUMAT and VUHARD subroutines developed in this study to accurately predict the flow stress of DMLS Ti6Al4V(ELI) was assessed from the statistical measures of correlation coefficient ($R^{2}$) and absolute average error (δ), based on the plot of Figure 8d, Figure 9d and Figure 10d. The correlation coefficient ($R^{2}$) can provide details on the strength of the linear relationship between the experimental and predicted values. However, the $R^{2}$-value may not reliably show better performance of the model, due to a tendency of the linear fit to be biased toward lower or higher values. This suggests that the $R^{2}$-value may be misleading if outlier values are present. The δ-value, on the other hand, is calculated through a term-by-term comparison of the relative error and is thus an unbiased statistical parameter for measuring the predictability of the model.

The standard statistical performance measures of $R^{2}$ and δ were obtained from the following expressions [42]:

(9) $$R^{2}=\frac{\sum_{i=1}^{N}{\left(E_{i}-\bar{E}\right)}^{2}\;{\left(P_{i}-\bar{P}\right)}^{2}}{\sum_{i=1}^{N}{\left(E_{i}-\bar{E}\right)}^{2\;}\sum_{i=1}^{N}{\left(P_{i}-\bar{P}\right)}^{2}}\;\mathrm{and}\;\delta =\frac{1}{N}\overset{N}{\underset{i=1}{\sum }}|\frac{E_{i}-P_{i}}{E_{i}}|$$

The correlation coefficients and average absolute errors obtained from these plots are summarised in Table 5.

It is evident from Table 5 that the implemented microstructural-based dislocation model in the VUMAT and VUHARD subroutines shows a very high degree of correlation as the $R^{2}$ values are above 0.97. It is also observed in this table that the absolute percentage errors between the numerical and the experimental values are all below 4%. These measures of correlation suggest that the numerical model developed in the present study accurately predicts the flow properties of the various microstructures of DMLS Ti6Al4V(ELI) tested here. The numerical model developed in this study is therefore suitable for use in designing the dynamic strength of DMLS Ti6Al4V(ELI) by controlling the morphology of its microstructure and the initial dislocation density present in the alloy.

### 7.3. Simulation of SHPB Tests Using the Johnson–Cook Model In-Built in ABAQUS

**Figure 11:**
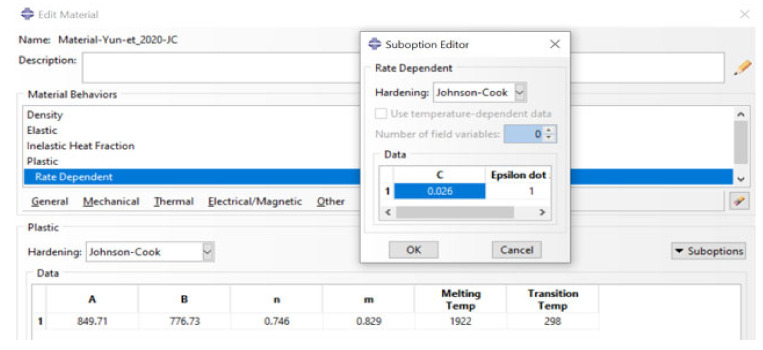
A typical material properties module for the J–C model in-built in ABAQUS.

The Johnson–Cook (J–C) model is a constitutive law that is commonly used to define isotropic flow properties of metals and metallic alloys during plastic deformation. This model is in-built in ABAQUS/CAE due to its simplicity of use. The model comprises an empirical form of the strain-hardening law, rate dependence and thermal softening and is normally of the form [36,63]:

(10) $$\sigma =\left(A+B\varepsilon_{pl}{}^{n}\right)\left(1+CIn{\frac{\dot{\varepsilon }}{\dot{\varepsilon }}}_{o}\right)\left(1-{\left(T^{*}\right)}^{m}\right)$$

where

(11) $$T^{*}=\left\{\begin{array}{l} \;\;\;0\;\;\;\;\;\;for\;T<T_{r}\\ \frac{T-T_{r}}{T_{m}-T_{r}}\;\;\;for\;T_{r}\leq T\;\leq T_{m}\\ \;\;1\;\;\;\;\;\;for\;T>T_{m}\; \end{array}\right.$$

where the parameters A, B, n, C and m are yield stress, strain-hardening factor, strain-hardening exponent, dimensionless strain rate hardening coefficient and thermal-softening exponent, respectively. The symbols $\varepsilon_{pl}$ and $\dot{\varepsilon }$ denote the equivalent plastic strain and strain rate, respectively, while ${\dot{\varepsilon }}_{o}$ is the reference strain rate at which parameters A, B and n are determined. This is usually a low strain rate (typically ≤ 1 s^−1^) where the effects of such strain rate on the plastic flow are negligible. Parameters T and $T_{m}$ are the test and melting temperatures of the materials, respectively, while $T_{r}$ is the room temperature. In this model, the transition temperature ($T_{t}$) is usually defined as a temperature at or below which there is no temperature-dependence of the yield stress and is usually taken as room temperature ($T_{r}$). This is the temperature used to determine the parameters A, B and n. As the model is in-built in ABAQUS for modelling isotropic flow properties, the user needs to provide the values of parameters A, B, n, m, $T_{m}$ and $T_{t}$ as part of metal plasticity material definition. The user also needs to provide the values of parameters C and ${\dot{\varepsilon }}_{o}$ when defining Johnson–Cook rate dependence. A typical properties module for the J–C model with rate dependence is shown in Figure 11. 

The J–C plasticity model is also extended for high-strain-rate transient dynamic applications where the temperature change (ΔT) in the model is generally computed internally by assuming adiabatic conditions using the following expression [63].

(12) $$\Delta T=\frac{\beta }{\rho ∁}\int \sigma \left(\varepsilon \right)\delta \varepsilon$$

Here the parameters β, ρ and ∁ are inelastic heat fraction (% of plastic work converted into heat and is normally taken as 0.9 for metal), density and specific heat capacity of a material, respectively. These three parameters must be provided for a simulation step that includes adiabatic heating effects.

The 3D finite element model of the SHPB test discussed in Section 6, which was used to investigate the high-strain-rate deformation behaviour of Ti6Al4V(ELI) samples, was modelled using the set of J–C parameters derived from literature and shown in Table 6. Beside these parameters, the values of specific heat, density and inelastic heat fraction 560 J/Kg·K, 4420 Kg/m^3^ and 0.9 were used [63,64] since the simulation was taken to include adiabatic heat effects. Interestingly, the sets of parameters in Table 6 differ considerably, even though they describe the behaviour of the same material, Ti6Al4V(ELI), though with different microstructures in certain cases.

**Figure 12:**
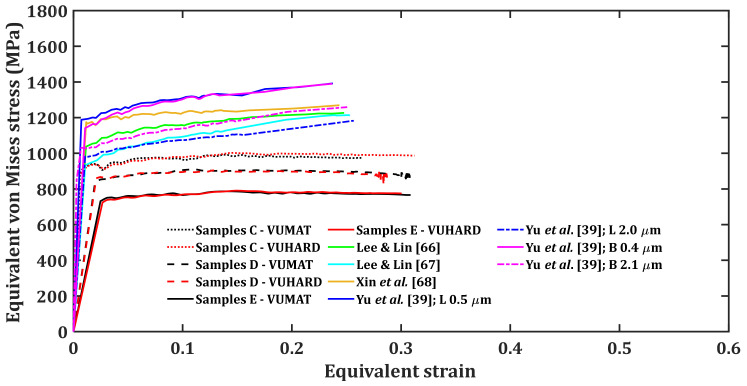
Results of SHPB test simulation at an impact velocity of 8 m/s and a temperature of 25 °C with the test samples modelled using various sets of J–C model parameters (note: B and L stand for bimodal and lamellar microstructures, respectively) and the numerical models developed in the present work.

**Figure 13:**
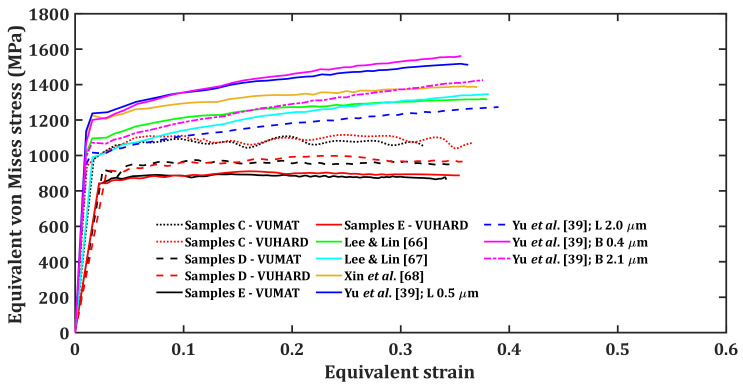
Results of SHPB test simulation at an impact velocity of 15 m/s and a temperature of 25 °C with the test samples modelled using various sets of J–C model parameters (note: B and L stand for bimodal and lamellar microstructures, respectively) and the numerical models developed in the present work.

**Figure 14:**
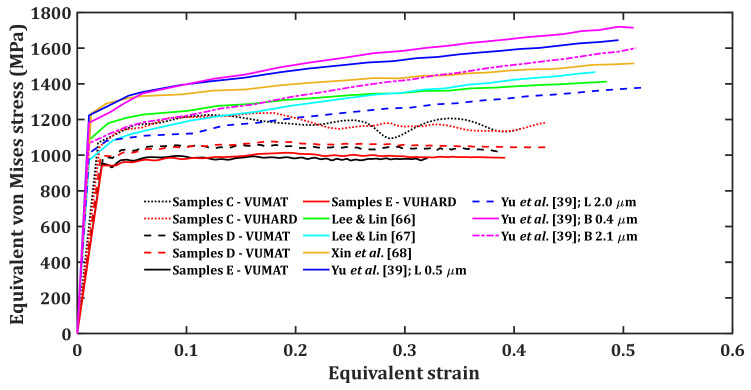
Results of SHPB test simulation at an impact velocity of 25 m/s and a temperature of 25 °C with the test samples modelled using various sets of J–C model parameters (note: B and L stand for bimodal and lamellar microstructures, respectively) and the numerical models developed in the present work.

As was the case for the numerical models developed in this work, three different striker velocities of 8 m/s, 15 m/s and 25 m/s, and a temperature of 25 °C were used to perform simulations and determine the equivalent von Mises stress and strain reported for the model at each striker velocity. Figure 12, Figure 13 and Figure 14 present the plots of the results obtained using different sets of J–C model parameters in Table 6 together with those from the numerical models developed in this study, using the VUMAT and VUHARD subroutines.

It is worth noting that the shape of the flow stress curve (relationship between the flow stress and plastic strain) seen in Figure 12, Figure 13 and Figure 14 for the J–C model is established empirically by isolating the effects of temperature and strain rate. The strain rate $\left(1+CIn\dot{\varepsilon }/{\dot{\varepsilon }}_{o}\right)$ and temperature $\left(1-{\left(T^{*}\right)}^{m}\right)$ parts are functions that scale the flow stress without necessarily influencing the shape of the flow stress curve. This implies that the J–C model is not adequate to represent flow characteristics of a material with the existence of recovery and recrystallization that allow stress to saturate as strain increases, as seen for samples C, D and E in this study. A similar observation was reported in the work of Yingnan et al. [65].

**Table 6:** A summary of J–C model parameters used to model the SHPB Ti6Al4V(ELI) samples.

J–C Model Parameter	Microstructure	α-Lath Size	A(MPa)	B(MPa)	*n*	C	*m*
Lee and Lin [66]	[-]	[-]	782.7	498.4	0.28	0.028	1
Lee and Lin [67]	[-]	[-]	724.7	683.1	0.47	0.035	1
Xin et al. [68]	[-]	[-]	920	380	0.578	0.042	0.633
Yu et al. [39]	Lamellar	0.5 μm	984.32	601.1	0.512	0.025	0.987
Yu et al. [39]	Lamellar	2.0 μm	829.30	524.32	0.621	0.024	0.715
Yu et al. [39]	Bimodal	0.4 μm	907.03	752.97	0.502	0.20	0.904
Yu et al. [39]	Bimodal	2.1 μm	849.71	776.73	0.742	0.026	0.829

The Taylor strain-hardening model that allows for strain-hardening and dynamic recovery as strain increases through calibrated parameters h and $k_{2}$ was used in the numerical models developed in the present research. This explains the flow stress saturation observed in samples C, D and E as opposed to continuous strain-hardening for the curves of the J–C model seen in Figure 12, Figure 13 and Figure 14.

It is very clear from these three figures that each set of J–C model parameters gives a different flow stress curve. The microstructural details of Ti6Al4V(ELI) from the work of Lee and Lin [66], Lee and Lin [67] and Xin et al. [68] are not inserted in Table 6 as they are not specified in these references. However, specific microstructural information of this alloy is available from the work of Yun et al. [39]. Each of these microstructures show different stress-strain curves in Figure 12, Figure 13 and Figure 14. For instance, lamellar and bimodal microstructures with average α-lath grain sizes of 0.5 µm and 0.4 µm, respectively, show higher values of flow stress in comparison with similar microstructures with average α-lath grain sizes of 2.0 µm and 2.1 µm, respectively. The lamellar microstructure with an average α-lath grain size of 2.0 µm shows lower values of flow stress in comparison to the bimodal microstructure with an average α-lath grain size slightly larger at 2.1 µm. The initial values of flow stress at lower strain for the lamellar microstructure with 0.5 µm α-lath average grain size are higher than those for the bimodal microstructure with a 0.4 µm α-lath average grain size. At higher strains, the lamellar microstructure with a 0.5 µm α-lath average grain size shows lower values of flow stress than those for the bimodal microstructure with a 0.4 µm α-lath average grain size, especially at the higher impact velocities of 15 m/s and 25 m/s in Figure 13 and Figure 14.

As seen in Figure 13 and Figure 14, at low values of strain and at striker velocities of 15 m/s and 25 m/s, the flow stress curves of samples type C (an average α-lath grain size of 2.5 µm) from the two subroutines developed here are close to those of Lee and Lin [67] and Yu et al. [39] for lamellar microstructure (average α-lath grain size of 2.0 µm). Whereas the flow stress curves of samples D and E, with much larger average α-lath grain sizes of 6 µm and 9 µm, respectively, are much lower than the rest, as seen in Figure 13 and Figure 14.

The preceding discussion suggests that no single set of J–C model parameters can be adequate in accurately describing the flow properties of any given microstructure of Ti6Al4V(ELI). This is mainly because each microstructure is related to a different set of J–C model parameters. In contrast, the microstructure- and dislocation-based constitutive numerical models presented in this study can be used to predict the flow properties of alloys, such as Ti6Al4V(ELI), that show a wide range of microstructures. The numerical models developed in the present study offer a few advantages including:

(a) The critical microstructural parameters of initial dislocation density and grain size are part of the few input parameters that are needed to adequately describe the flow stress.
(b) Both strain-hardening and dynamic recovery that occur for deformation at high temperatures and high strain rates are articulated in these numerical models through calibrated parameters h and $k_{2}$. These parameters are insensitive to the different microstructures of samples C, D and E, as opposed to the case of the J-C model where its many parameters vary with microstructure.
(c) There are only four input parameters that are influenced by the microstructure in the numerical models developed in this study. These include initial dislocation density and grain size, which are determined directly from the microstructure, as well as two viscous drag stress-fitting parameters (χ  and ζ) determined empirically from the experimental data. This is less than the five microstructure-sensitive parameters of the J–C model that are empirically determined from the experimental data.

## 8. Conclusions

Three dimensional SHPB numerical tests were simulated in this paper with the test specimens modelled using the VUHARD and VUMAT subroutines developed in the present study. The numerical results from these simulations and those obtained from experimental testing at the high strain rates of 750 s^−1^, 1500 s^−1^ and 2450 s^−1^ and at temperatures of 25 °C, 200 °C and 500 °C were compared for sample types C, D and E. The SHPB tests of Ti6Al4V(ELI) were further modelled with various sets of J–C model parameters available in literature. The following conclusions were deduced from this work:

- The numerical and experimental results showed a high degree of correlation with correlation coefficients ($R^{2}$) above 0.97.
- The absolute percentage error between the numerical and experimental results were for both subroutines and for all samples below 4%, which is acceptably low.
- The results of the simulation using different sets of J–C model parameters, displayed a wide variety of stress-strain curves, with each set of parameters showing a different curve.
- The wide range of microstructures associated with the Ti6Al4V(ELI) alloy were found to limit the utility of the J–C model to accurately describe and simulate the flow properties of various forms of the alloy.
- The numerical models developed here were found to have advantage over the J–C model in that, unlike the J–C model, the critical parameters of microstructures such as grain size and dislocation density that influence the flow stress form part of the numerical model inputs.
- The numerical models developed in this study were found to give good predictions of the strain-hardening and dynamic recovery processes that prevail for deformations at high strain rates and high temperature, which is not the case for the J–C model.

Future studies should aim at testing the performance of the VUHARD and VUMAT subroutines developed in this study in other complex dynamic simulations such as the Taylor impact test and blasting loading of plates. The microstructure-sensitive numerical models developed in this study should be extended to include some form of failure criterion or damage model to incorporate unloading and damage evolution.

## Figures and Tables

**Table 1:** Composition of Ti6Al4V(ELI) alloy powder as supplied by TLS Technik GmbH and that of ASTM F3001-14.

Element	Al	V	Fe	O	C	N	H	Ti
(wt.%)	6.34	3.944	0.25	0.082	0.006	0.006	0.00	Bal.
ASTM F300-14	5.50–6.50	3.50–4.50	<0.25	<0.13	<0.08	<0.05	<0.012	Bal.

**Table 2:** Microstructural features, model input parameters for DMLS Ti6Al4V (ELI) alloy [44,53,54].

Samples Type	Average Grain Size (μm)	$\mathrm{Average}\;\mathrm{Initial}\;\mathrm{Dislocation}\;\mathrm{Density}\;(m^{-2})$
C	2.5	5.73 × 10^14^
D	6.0	5.09 × 10^14^
E	9.0	7.00 × 10^14^

**Table 3:** Calibrated model input parameters and other constants obtained from the literature.

Prescribed Parameters	Value and Units	Ref.	Fitted Parameters	Values and Units
$\mathrm{Boltzmann}\;\mathrm{constant}\;(k_{b}$)	1.38 × 10^−23^ m^2^kgs^−2^k^−1^	-	$\sigma^{o}$	1063.2 MPa
Burgers vector (*b*)	$2.95\times 10^{-10}\;m$	[55]	$g_{0i}$	0.25
$\mathrm{Reference}\;\mathrm{strain}\;\mathrm{rate}\;({\dot{\varepsilon }}_{o})$	10^7^ s^−1^	[56]	h	8.3 × 10^15^ m^−2^
$K_{H-P}$	$0.328\;{\mathrm{MPam}}^{1/2}$	[57]	$k_{2}$	10
P	1	[58]	*ζ*	(MPa)
Q	2	[58]	Samples C	207
Taylor factor (M)	3	[59]	Samples D	210
α	0.2	[60]	Samples E	210
μ	$49.02-5.821/\left(e^{\frac{181}{T}}-1\right)\left(\mathrm{GPa}\right)$		χ	
		[58]	Samples C	0.00032
			Samples D	0.00030
			Samples E	0.00020

**Table 4:** Computation time for different mesh sizes.

Subroutine	VUHARD	VUMAT
Mesh size (mm)	Time (s)	Time (s)
1	195.4	216.7
0.5	515.6	555.8
0.25	979.6	997.3
0.18	1627.1	1692.9

**Table 5:** Absolute percentage error (*δ*) and correlation coefficient ($R^{2}$) between the experimental and numerical model values of flow stress for various forms of DMLS Ti6Al4V(ELI).

Measure	*R* ^2^				*δ* (%)			
Strain	0.1		0.2		0.1		0.2	
Samples	VUMAT	VUHARD	VUMAT	VUHARD	VUMAT	VUHARD	VUMAT	VUHARD
C	0.993	0.982	0.978	0.983	2.54	3.73	2.74	3.01
D	0.994	0.987	0.995	0.994	1.77	2.66	1.89	2.44
E	0.978	0.981	0989	0.982	3.18	3.22	1.28	2.77

## Data Availability

The datasets presented in this study are available on request from the corresponding author, and are not publicly available as they form part of an ongoing research.
